# Peptide Carrier-Mediated Non-Covalent Delivery of Unmodified Cisplatin, Methotrexate and Other Agents via Intravenous Route to the Brain

**DOI:** 10.1371/journal.pone.0097655

**Published:** 2014-05-21

**Authors:** Gobinda Sarkar, Geoffry L. Curran, Jann N. Sarkaria, Val J. Lowe, Robert B. Jenkins

**Affiliations:** 1 Department of Experimental Pathology, Mayo Clinic and Foundation, Rochester, Minnesota, United States of America; 2 Department of Neurology, Mayo Clinic and Foundation, Rochester, Minnesota, United States of America; 3 Department of Radiation Oncology, Mayo Clinic and Foundation, Rochester, Minnesota, United States of America; 4 Department of Nuclear Medicine, Mayo Clinic and Foundation, Rochester, Minnesota, United States of America; University Hospital of Navarra, Spain

## Abstract

**Background:**

Rapid pre-clinical evaluation of chemotherapeutic agents against brain cancers and other neurological disorders remains largely unattained due to the presence of the blood-brain barrier (BBB), which limits transport of most therapeutic compounds to the brain. A synthetic peptide carrier, K16ApoE, was previously developed that enabled transport of target proteins to the brain by mimicking a ligand-receptor system. The peptide carrier was found to generate transient BBB permeability, which was utilized for non-covalent delivery of cisplatin, methotrexate and other compounds to the brain.

**Approach:**

Brain delivery of the chemotherapeutics and other agents was achieved either by injecting the carrier peptide and the drugs separately or as a mixture, to the femoral vein. A modification of the method comprised injection of K16ApoE pre-mixed with cetuximab, followed by injection of a ‘small-molecule’ drug.

**Principal findings:**

Seven-of-seven different small molecules were successfully delivered to the brain via K16ApoE. Depending on the method, brain uptake with K16ApoE was 0.72–1.1% for cisplatin and 0.58–0.92% for methotrexate (34-50-fold and 54–92 fold greater for cisplatin and methotrexate, respectively, with K16ApoE than without). Visually intense brain-uptake of Evans Blue, Light Green SF and Crocein scarlet was also achieved. Direct intracranial injection of EB show locally restricted distribution of the dye in the brain, whereas K16ApoE-mediated intravenous injection of EB resulted in the distribution of the dye throughout the brain. Experiments with insulin suggest that ligand-receptor signaling intrinsic to the BBB provides a natural means for passive transport of some molecules across the BBB.

**Significance:**

The results suggest that the carrier peptide can non-covalently transport various chemotherapeutic agents to the brain. Thus, the method offers an avenue for pre-clinical evaluation of various small and large therapeutic molecules against brain tumors and other neurological disorders.

## Introduction

The blood-brain barrier (BBB) severely inhibits the ability to deliver therapeutics to the brain. Indeed, it has been reported that >98% of potential drugs having molecular weights of even <500 Daltons cannot reach the brain because of the BBB [1], [2]. Existing methods for delivering drugs to the brain (e.g. convection-enhanced delivery (CED) [3], [4], ultrasound-mediated delivery [5]) suffer from several limitations: they can be very invasive, they can compromise drug efficacy; and/or they can cause irreversible damage to the brain [6]–[8]. Thus, there is a great need for methods that can deliver drugs to the brain while reducing or eliminating these limitations.

Since the BBB poses a serious obstacle to delivering therapeutics to the brain, a damaged BBB associated with brain tumors provides a common avenue for delivering chemotherapeutics. However, the BBB is only marginally disrupted in grade 2 and 3 gliomas. Furthermore, in grade 4 gliomas the BBB damage is limited to the area of vascular damage. In all gliomas neoplastic tumor cells have widely invaded well beyond the region of obvious radiologic involvement. Thus it has been argued that novel methods are urgently needed that can enhance drug delivery throughout the brain beyond the level obtained via a damaged BBB [9].

The BBB harbors receptors that allow transport of cognate protein ligands from the vasculature to the brain through transcytosis [10], [11]. Several investigators have utilized such ligand-receptor systems to develop strategies for delivering various proteins to the brain. However, all these methods employ *covalent linking* of the target proteins to a peptide carrier comprised of the receptor-binding domain of a ligand [12], [13], an antibody against a receptor [14]–[16] or to other peptides and proteins deemed to have BBB transport activity [17], [18]. Covalent linking of a carrier entity to a protein ‘load’ involves complex issues such as expertise in linkage chemistry, necessity of purification after linkage, evaluation of functionality after purification etc. Incorporating a given drug into BBB-penetrating nanoparticles also requires considerable efforts to formulate the nanoparticles harboring the drug of choice and a separate method such as CED to deliver the nanoparticles across the BBB [19], [20]. Consequently, we sought to develop non-covalent brain delivery methods of therapeutic agents that would avoid these limitations. We have recently reported creation of a carrier peptide (termed K16ApoE) that transported various proteins and immunoglobulins across the BBB in a non-covalent manner [21]. Since cancer therapeutics comprise both large and small-molecule agents, we explored if the carrier peptide would also enable non-covalent delivery of ‘small molecules’ to the brain.

Based on our previous work [21] we hypothesized that the ApoE-like protein-K16ApoE complex causes conformational change of LDLR-expressing cells at the BBB creating transient pores through which passive transport of other (non-ligand) molecules to the brain can take place. We extend our hypothesis to include the possibility that normal ligand-receptor interactions at the BBB also create transient pores that allow some non-ligand molecules to passively cross the barrier. We have tested these hypotheses in the context of delivering methotrexate (MW 454.4), cisplatin (MW 300.06), Evans Blue (MW960.81), Crocein Scarlet (MW 584.54), Light green SF (MW 792.88), a synthetic 8-amino acid peptide, Y8 (MW 1323) and I-125 to the brain. Our results appear to support the above hypotheses, and illustrate a novel approach to modulate the BBB for systemic delivery of ‘drug-size’ chemotherapeutics and radioisotopes to the brain in a non-covalent manner.

## Materials and Methods

### Materials

*Ethics Statement*: This study was carried out in strict accordance with the recommendations in the Guide for the Care and Use of Laboratory Animals of the National Institutes of Health. The protocol was approved by the Institutional Animal Care and Use Committee (IACUC) of the Mayo Clinic (Protocol number A55911)). All surgery was performed under sodium pentobarbital anesthesia, and all efforts were made to minimize suffering.

Only female mice (B6SJLF1) purchased from the Jackson Laboratories were used. Evans Blue was obtained from Fisher Scientific (catalog# E515),Crocein Scarlet was from Bio Rad (catalog # 161-0417) and Light Green SF was obtained from Sigma-Aldrich (catalog # L1886).

Synthesis of the peptides used was carried out at the Mayo Proteomic Core Facility. The transporter peptide K16ApoE, has the following amino acid sequence (in single-letter code): KKKK KKKK KKKK KKKK LRVR LASH LRKL RKRL LRDA, this peptide had NH2 group at both ends.

### Delivery of Various Dyes and other Molecules to the Brain

The femoral vein was catheterized as follows: the medial surface of the left hind limb was first shaved and sterilized with Betadine. A 2-cm incision was made along the mid line of the medial surface of the limb. The skin and muscles were retracted to expose the femoral vein. The vein was catheterized with PE50 polyethylene catheter heat tapered to PE10. The femoral vein was secured with three ligatures as follows: one ligature supported the catheter with attachment to muscle tissue laterally, a second ligature supported the catheter with the femoral vein, and the third ligature was placed medially at the point where the venous catheter was introduced into the femoral vein. The carrier peptide (or other peptides) was first injected through the catheter, the dyes and other small molecules were injected through the same catheter ten minutes after injecting the carrier peptide. In some experiments, the carrier peptide and other molecules such as cisplatin and methotrexate were first mixed and then injected. At the completion of the experiment, the animal was sacrificed with an overdose of sodium pentobarbital (200 mg/Kg, IP ). Each animal was then transcardially perfused with PBS followed by perfusion with 10% neutral buffered formalin, and half the brain was processed for analysis.

### Brain Imaging by Micro Single Photon Emission Computed Tomography (microSPECT)

Imaging by micro SPECT was conducted on a Gamma Medica X SPECT System (GE Healthcare) [22]. Radiolabeled ^I-125^peptide (Y8) or free I-125 was injected 10 minute after injection of the carrier peptide alone or after injection of the carrier peptide mixed with cetuximab or after injection of insulin through the use of a catheter in the femoral vein. After 1 h, each mouse was euthanized and the systemic blood supply was transcardially perfused with 10 ml of phosphate buffered saline, followed by imaging.

### Quantification of Cisplatin in Brain

Fresh or frozen brain hemispheres were weighed and then placed in 10 ml glass vials. 2 ml of 70% nitric acid was then added and heated at 120°C to dryness; the process taking ∼2 h. This step was repeated once. One ml of 30% hydrogen peroxide was then added, followed by heating at 120°C to dryness; the process taking ∼1 h. This step was also repeated once. Finally, the digested product was dissolved in 1 ml of 1% nitric acid. Brain specimens digested this way were sent to the Mayo Clinic Metals Laboratory for quantification of cisplatin by Atomic Absorption Spectrometry.

### Quantification of Methotrexate in the Brain

Fresh or frozen brain specimens were sent to the Mayo Clinic Drug and Toxicology laboratory which offers quantification of methotrexate for clinical specimens. Briefly, the method involves weighing the brain followed by homogenization in water. A fixed amount of an internal methotrexate standard is then added to an aliquot of the brain homogenate, and the total amount of methotrexate is then extracted and reconstituted in a reconstitution solution. An aliquot of the reconstituted methotrexate is then analyzed and quantified through LC-MS/MS.

### Evaluation of Permeabilization of the BBB by Insulin

Various amounts of insulin (Humulin) were injected into the femoral vein through catheter placed as described earlier. I-125 or other molecules were then injected 10 min after injecting insulin. MicroSPECT imaging was done on post-perfused mice brain as described earlier to quantify amount of I-125 uptake by the brain.

### Intracranial Injection of Evans Blue

A Stoelting stereotaxic frame fitted with a custom fit anesthesia inhalation system was devised to deliver 1.5–2% isoflurane with 2 L/min oxygen through the custom fit nose cover. Hair was clipped from the head and the skin was decontaminated with betadine. Ocular lubricant (Artificial Tears) was applied to prevent drying of the eyes. The mouse head was stabilized in the frame using ear bars. A 6 mm midsagittal incision was completed. The following coordinates were located: 1) 2.0 mm posterior to bregma, 2) 2.0 mm lateral to midline, 3) 3.0 mm ventral to the surface of the skull. A hole was drilled into the skull using a frame attached Series SR Foredom drill, but did not penetrate the dura. Delivery of Evans Blue was accomplished by using a pre-loaded microinjection syringe (fitted with a 30-gauge needle) attached to the microinjection device holder on the frame. The 30-gauge needle was slowly lowered to 3 mm and the predetermined volume of Evans Blue was injected over 3 min. After injection the needle remained at the predetermined depth for 2 min and then subsequently removed slowly. The skin incision was closed with 3-0 vicryl suture. Each mouse was euthanized at 1 hr post-surgery. The mouse was transcardially perfused with 10 ml phosphate buffered saline pH 7.4.

## Results

### The Peptide Transporter K16ApoE, can Transport Evans Blue Non-covalently in a Dose-dependent Manner

**Figure 1:**
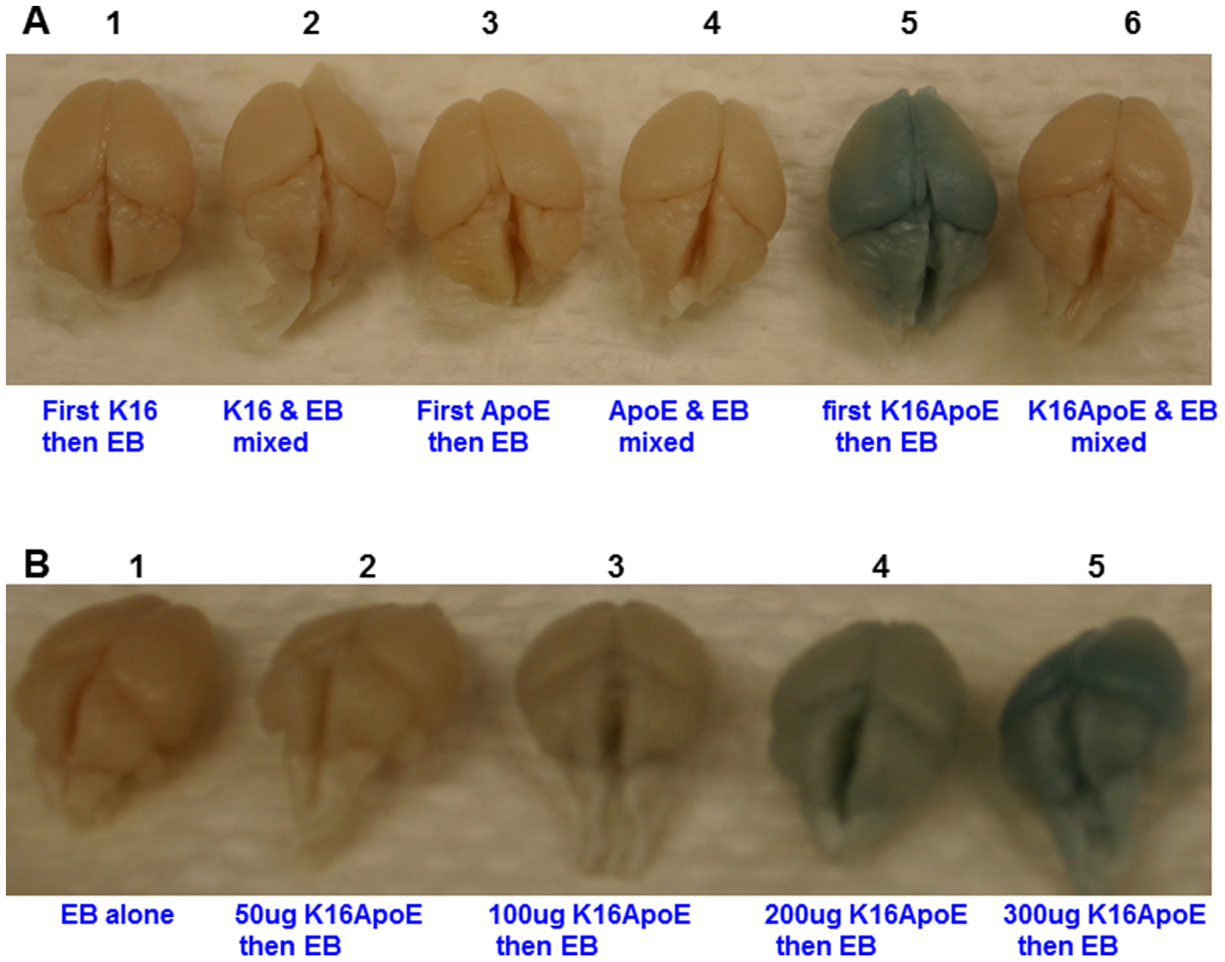
Evaluation of brain delivery of Evans Blue (EB) by various peptides (A) and by different concentration of peptide K16ApoE (B). 67.5 picomoles of each of the peptides (K16, ApoE and K16ApoE) was either injected first followed in 10 min by injection of EB (40 ul of a 2% solution) or the dye and the peptides were mixed together and then injected. Mice were perfused with saline 2 h after injection and then brains were collected for visualization.

We previously observed that intra-venous injection of free K16ApoE resulted in transient delivery of beta-galactosidase across the BBB [21]. This observation led us to hypothesize that such transient permeabilization of the BBB by the carrier peptide K16ApoE should allow passive transport of other molecules to the brain. We also hypothesized that molecules smaller in size than beta-galactosidase delivered in this manner would have enhanced passive transport to the brain. To test these hypotheses, we have first evaluated passive non-covalent transport of Evans Blue (EB, MW 960.81) to the brain with prior injection of free K16ApoE or other control peptides. In this experiment, EB was injected after injection of either K16 (a peptide consisting of 16 lysine residues only), ApoE (a 20-aa peptide consisting of the LDLR-binding domain of ApoE), K16ApoE (a 36-aa peptide consisting of the K16 and ApoE peptides) or mixed with each of these peptides and then injected. Visual inspection of the results presented in Figure 1A show that a visible amount of the dye crossed the BBB when the dye was injected after K16ApoE. It is interesting to note that there was no brain-uptake of the dye when K16ApoE and the dye were injected together. Results presented in Figure 1B show that increasing amounts of the dye was transported to the brain with increasing amounts of K16ApoE. These results provide strong evidence that by itself K16ApoE can allow passive delivery of EB to the brain in a non-covalent manner.

### K16ApoE Allows other Dye Molecules to be Transported to the Brain Besides Evans Blue

**Figure 2:**
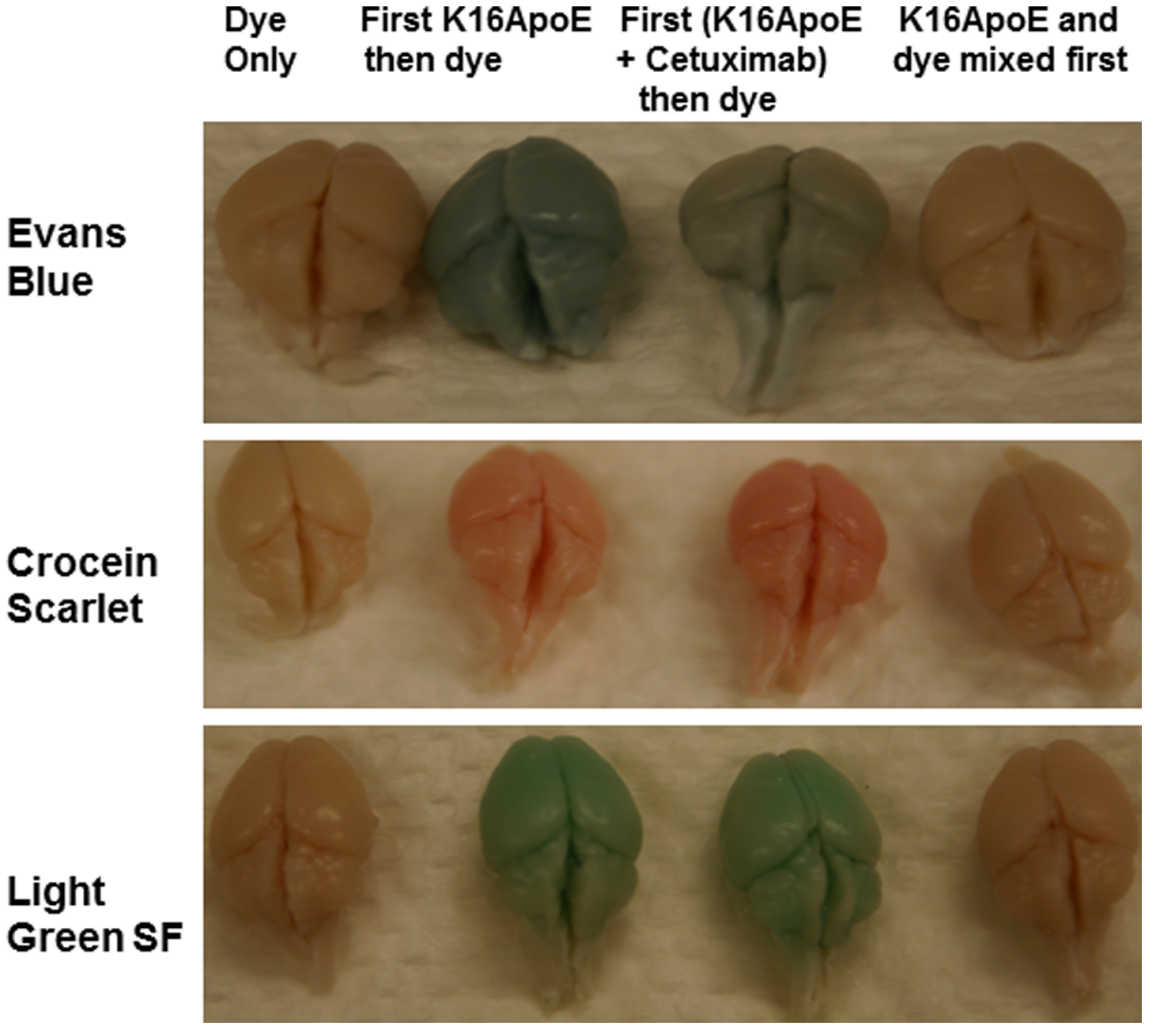
K16ApoE-mediated brain delivery of blue (EB), red (Crocein Scarlet) and green (Light Green SF) dyes to the brain. Three different approaches were assessed for dye delivery: 1. K16ApoE was injected first then a given dye was injected 10 min after (second columns of brain specimens); 2. K16ApoE was mixed with 300 ug of cetuximab and injected followed by injection of a given dye 10 min after 3^rd^ column of brain specimens), and 3. K16ApoE and the dyes were mixed and injected (fourth column of brain specimens). The first column of brain specimens represents animals receiving injection of a given dye alone. Mice were perfused with saline 2 h after injection and then brains were collected for visualization. 67.5 picomole of K16ApoE was used in each experiment. 40 ul of a 2% solution of each of the dyes were used for injection into a 20 g mouse (amount of dye injected varied accordingly with weight of mice).

To evaluate if K16ApoE would enable delivery of other molecules to the brain besides EB, we attempted to deliver Crocein Scarlet (MW 584.54) and Light Green SF (MW 792.88) to the brain. In addition to using K16ApoE alone, an alternative strategy was also employed that took advantage of the protein carrying ability of the peptide. This strategy involved mixing K16ApoE with a therapeutic protein (in this instance cetuximab - a monoclonal antibody against EGFR), injecting this mixture, and then injecting the dyes. (Figure S1 demonstrates that K16ApoE mediates brain uptake of cetuximab when the two were first mixed and then injected). Results presented in Figure 2 demonstrate that both the original and the alternative strategies can successfully deliver the dye molecules to the brain. Collectively, these results provide strong evidence for the generality of our method for delivering various ‘drug-size’ molecules to the brain in a non-covalent manner. Brain-uptake of radiolabeled cetuximab was evaluated by microSPECT (Figure S1).

### Opening of the BBB by K16ApoE is Transient but EB Delivered via the Peptide Remains in the Brain for a Long Time

Transient opening of the BBB is required for all approaches that attempt to deliver therapeutic agents to the brain. However, to minimize potential toxicity, the duration of BBB permeability must be limited. Limiting the duration of permeability should also facilitate retention of the agent(s). Thus we investigated the duration of permeability and how long molecules remained in the brain.

**Figure 3:**
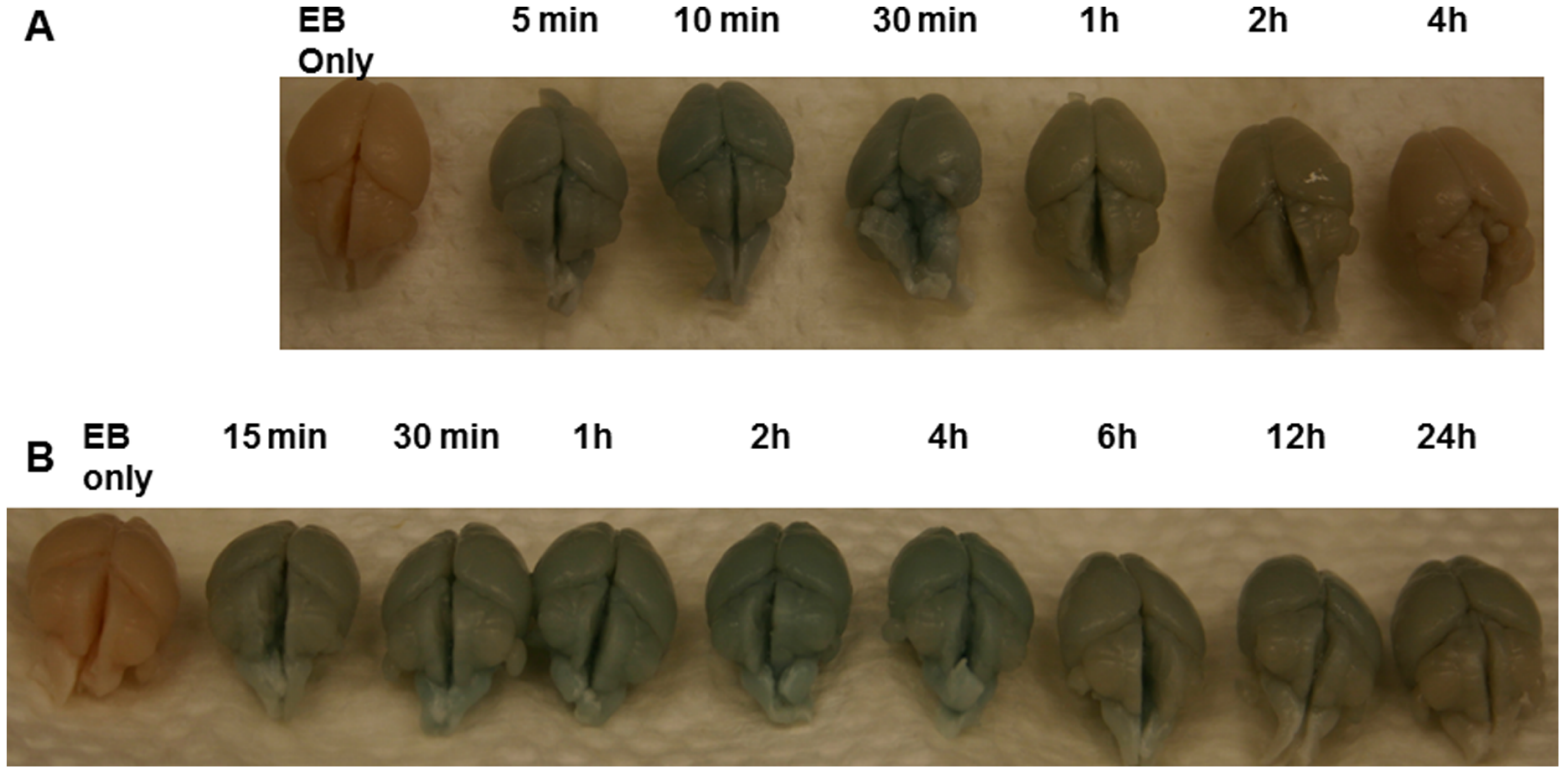
Evaluation of time-frame of BBB permeabilization by K16ApoE and retention-time of EB in the brain delivered by K16ApoE. A- Stains of EB from brain specimens from mice in which K16ApoE was injected first and then EB was injected 5 min, 10 min, 30 min, 1 h, 2 h and 4 h after. Cardiac perfusion was done 1 h after injection of the dye followed by collection of brains. B - Stains of EB from brain specimens from mice in which K16ApoE was injected first and then EB was injected 10 min after. Perfusion was done at indicated times followed by collection of brains.

To ascertain the length of time the BBB remains open after IV administration of K16ApoE, we injected EB from five minutes to 4 h after the injection of the peptide. The intensity of the staining of the brain specimens indicates that the BBB remains permeable for up to 30 min, after which it gradually reverts to normal (Figure 3A). The length of time the BBB remains open after administration of K16ApoE allows an appropriate time-frame for administration of a given drug after injection of the peptide.

To assess the length of time the dye remains within the brain after being delivered by our K16ApoE-mediated method, we injected the peptide followed by EB 10 min later. Brain specimens were collected at different times from 15 min to 24 h after injection of the dye. Visual inspection of the results presented in Figure 3B indicate that a considerable amount of EB delivered in this manner remains in the brain for up to 4 h and lesser amount for up to 24 h. These results imply that a potential drug molecule the size of EB (MW 960.81) delivered to the brain via our method will have a reasonable time-frame to exert its clinical effect.

### Delivery and Quantification of Cisplatin, Methotrexate and a Synthetic Peptide Y8 to the Brain via K16ApoE

We explored the delivery of cisplatin (MW 300.06) and methotrexate (MW 454.06) to the brain via K16ApoE for three reasons: First, they are well-established chemotherapeutic agents [23], [24]; second, they have in vitro efficacy against glioma [25], [26]; and third these drugs poorly cross the BBB [27], [28].

We explored three different but related methods to accomplish K16ApoE-mediated brain uptake of cisplatin and methotrexate. In the first, K16ApoE was injected first and then cisplatin or methotrexate was injected 10 min later (method 1). In the second, a mixture of K16ApoE and cetuximab were mixed and injected followed by cisplatin or methotrexate 10 min later (method 2). The third involved one injection of a mixture of K16ApoE with cisplatin or methotrexate (method 3).

Results presented in Table 1 show that all three K16ApoE-mediated approaches allow transport of both cisplatin and methotrexate to the brain. Uptake of cisplatin to the brain appears to be about 0.72–1.14% of the injected dose depending on the method, which is 34-53-fold greater with K16ApoE compared to brain-uptake of cisplatin injected alone. Interestingly, the results also show that comparable brain-uptake of cisplatin occurs irrespective of whether the drug is administered separately (39-fold over cisplatin alone) from K16ApoE or mixed with it (33-fold over cisplatin alone). K16ApoE-mediated brain uptake of methotrexate was 0.54 to 0.92% of the injected dose, which was 54 to 92-fold greater with the carrier peptide than without. Thus, K16ApoE-mediated brain uptake of cisplatin and methotrexate appears to be comparable.

**Table 1:** Quantification of K16ApoE-mediated brain-uptake of cisplatin (Cp) and methotrexate (MTX).

Brain uptake of cisplatin (Cp)			
Experimental group	Brain Cp level (Mean ± SD)	Fold change	% delivery
Group 1	64.66±19.21 ng		0.02
Group 2	2557±421.4 ng	39	0.86
Group 3	3417.66±843.01 ng	53	1.14
Group 4	2178±1789.95 ng	34	0.72
**Brain uptake of methotrexate (MTX)**			
**Experimental group**	**Brain MTX level (Mean ± SD)**	**Fold change**	**% delivery**
Group 1	22.42±2.26 ng		0.01
Group 2	2745.01±2070.91 ng	92	0.92
Group 3	1618.65±1037.77 ng	54	0.54
Group 4	1735.43±2007.19 ng	58	0.58

300 ug of the carrier peptide K16ApoE, 300 ug of cetuximab and 300 ug of cisplatin (Cp) were used in this experiment. Group 1- these animals received only Cp or MTX. Group 2- these animals received injection of K16ApoE then injection of either Cp or MTX. Group 3- these animals received an injection of K16ApoE mixed with cetuximab, followed by an injection of Cp or MTX. Group 4- these animals received an injection of K16ApoE mixed with Cp or MTX. Post-perfused brains were collected after 1 h of final injection and processed for respective assays. Fold change for Group 2 has been obtained by dividing the mean value for Group 2 by the mean value for group 1; fold change for Group 3 has been obtained by dividing the mean value for this group by the mean value of Group 1, and so on. ‘% delivery’ indicates the fraction of Cp or MTX in brain compared to the injected dose. Six animals in each group have been used.

**Figure 4:**
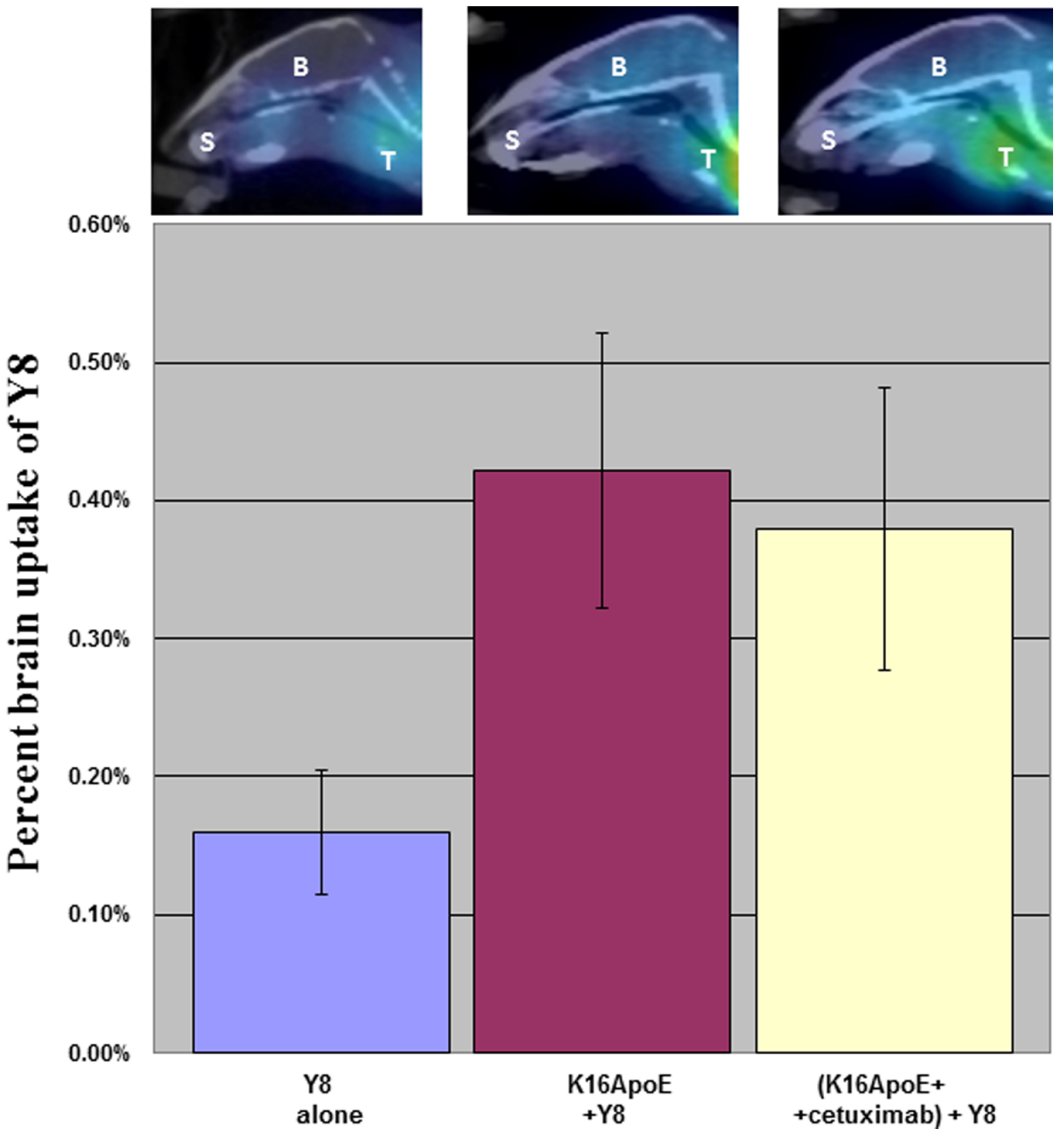
Brain imaging and quantification (via microSPECT) of brain-uptake of Y8 delivered via K16ApoE. ^I-125^labeled Y8 was delivered to the brain by first injecting K16ApoE then injecting Y8 as described earlier. Brain delivery of Y8 was also assessed after injection of a mixture of K16ApoE and cetuximab (300 ug of each). A. The bars represent brain uptake of ^I-125^labeled Y8 when injected by itself (blue), injected after injection of K16ApoE (majenta) or injected after injection of a mixture of K16ApoE and cetuximab (yellow). Six mice were evaluated for each group. Images of brains by microSPECT representing delivery of ^I-125^labeled Y8 at different conditions are presented on top of the bars. Brain images were taken after cardiac perfusion with saline. T- thyroid gland; S – salivary gland; B- brain.

We also quantified brain uptake of a short synthetic peptide consisting of eight tyrosine residues (Y8, MW 1323). Y8 was radio-labeled and uptake quantitated by micro-single photon emission computed tomography (micro-SPECT). Brain-uptake of ^I-125^Y8 was measured after prior injection of either K16ApoE or a mixture of K16ApoE and cetuximab. Figure 4A shows that >2.5-fold of Y8 was transported to the brain by either method compared to transport of Y8 by itself. MicroSPECT images of brain specimens presented in Figure 4B provide additional evidence of enhanced brain-uptake of Y8 by K16ApoE.

### Administration of Insulin Enhances Brain-uptake of I-125

One conceptual extrapolation of the preceding results is that normal ligand-receptor interactions intrinsic to the BBB may routinely allow passage of additional non-ligand molecules across the barrier. To test this hypothesis, we evaluated BBB permeability after administration of insulin, a ligand having cognate receptors/transporters on the BBB [29]. Experiments to visually assess brain-uptake of EB via administration of insulin did not show any transport of the dye to the brain. Assuming there is a size limitation of molecules permitted to cross the BBB through transient pores created by a particular ligand-receptor interaction, we decided to assess the permeability of cisplatin, a molecule smaller in size than EB. Three different concentrations of insulin were used: 250 ug, 500 ug and 1000 ug. No increase in brain-uptake of cisplatin was observed at 250 and 500 ug insulin administration. However, ∼18% more cisplatin in brain was observed at 1000 ug of insulin compared to administration of cisplatin alone, but the result was not statistically significant (data not shown).

**Figure 5:**
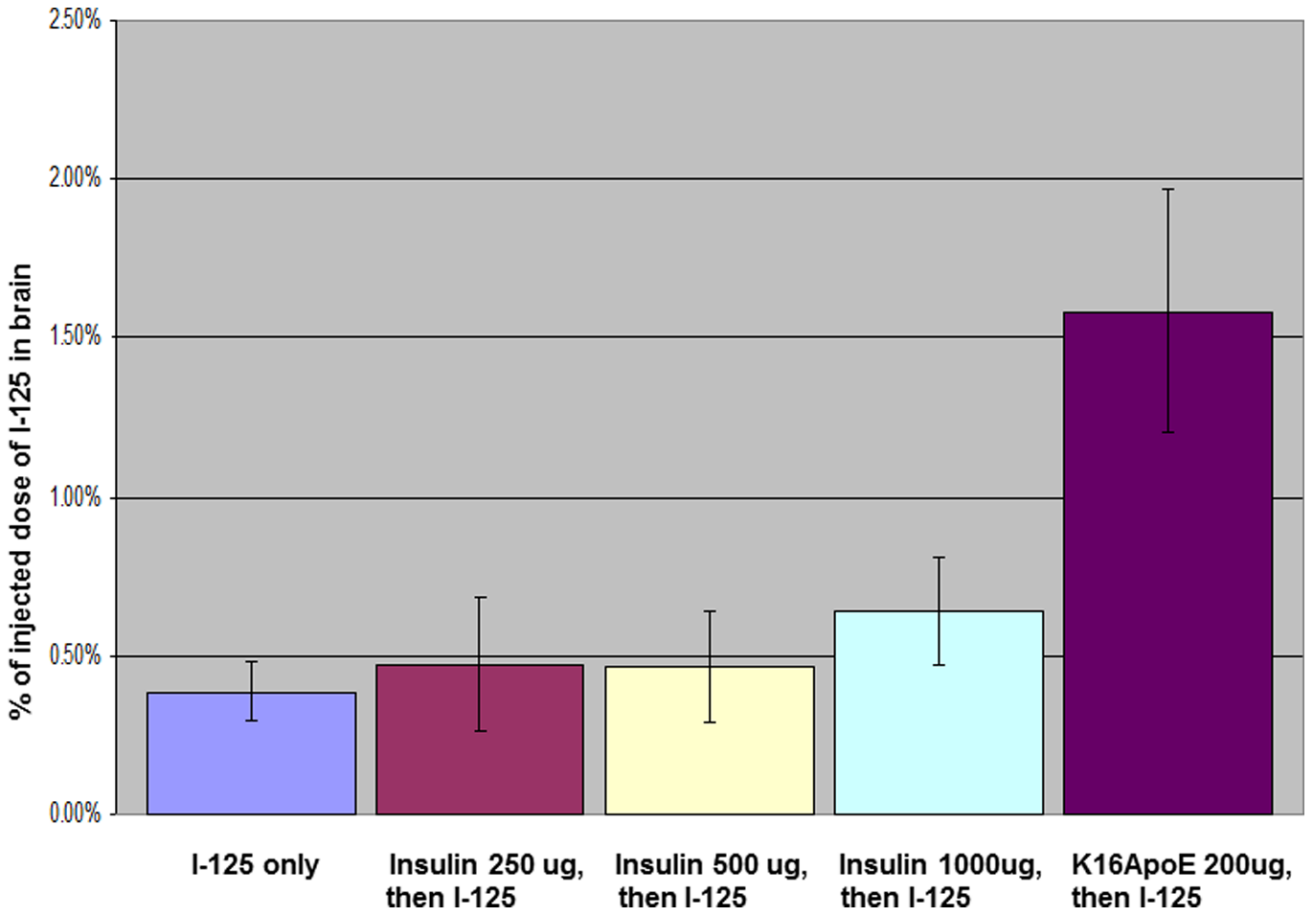
Brain imaging and quantification (via microSPECT) of brain-uptake of I-125 via insulin injection. I-125 was injected 10 min after injection of 250 ug, 500 ug and 1000 ug of insulin, respectively, and 200 ug of K16ApoE. Quantification of I-125 in the brain was done after cardiac perfusion. Six mice were evaluated for each group.

Next, we evaluated if administration of insulin modulates brain-uptake of I-125, a molecule much smaller than either EB or cisplatin. There was no significant increase in the uptake of I-125 after administration of 250 ug and 500 ug of insulin (Figure 5). However, there was 61% more brain-uptake of I-125 when I-125 was injected after administration of 1000 ug of insulin; this increase in brain-uptake of I-125 appeared to be statistically significant (p<0.05). It is noteworthy that brain-uptake of I-125 was ∼400% greater when injected with K16ApoE compared to administration of I-125 alone (p<0.00002).

### Brain Distribution of Evans Blue (EB) via K16ApoE-mediated Intravenous Injection is Global but is Localized via Intracranial Injection

**Figure 6:**
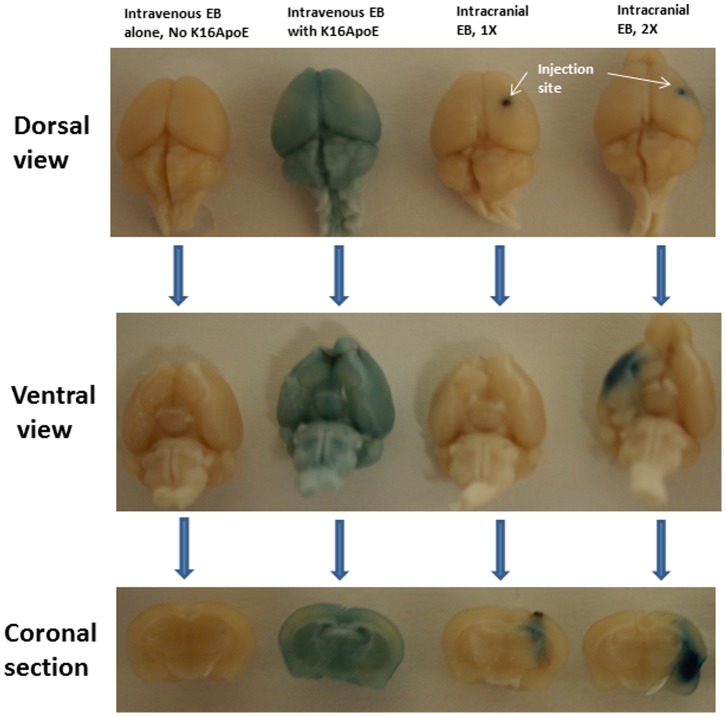
Qualitative comparison of brain uptake of Evans Blue (EB) via direct intracranial and K16ApoE-mediated intravenous injections. Intravenous injection through femoral vein involved delivery of 1mg of EB with or without K16ApoE. Assuming (based on several experiments and results from delivery of cisplatin and methotrexate presented in this paper) 1% of the intravenously-injected molecules reaches into the brain (when delivered via K16ApoE), 10 ug (1X in the Figure) and 20 ug (2X in the Figure) of EB were used for intracranial injection. All animals used in the experiment underwent cardiac perfusion 1 h after delivery of the dye by either method, after which brains were collected and photographed before and after coronal sections were made.

In many situations, intracranial injection is employed to administer various drugs into the brain. To be acceptable as a realistic drug-delivery method, brain distribution of a drug delivered via K16ApoE-mediated intravenous route should be comparable to that obtained by intracranial injection. To explore a visual comparison of brain distribution of Evans Blue (EB) by direct intracranial injection and by K16ApoE-mediated intravenous injection into the femoral vein, EB was delivered to the brain by both methods. Brains were collected after cardiac perfusion with saline. Photographs of whole brains and half-brains obtained after coronal sections were taken. The results presented in Figure 6, especially after coronal section, clearly show that whereas brain distribution of the dye remain localized after intracranial delivery, it is distributed throughout the brain when delivered via K16ApoE-mediated femoral vein injection.

## Discussion

Currently, several strategies have been developed that overcome the restriction imposed by the BBB for delivering therapeutic agents to the brain. In general, these methods rely on physical and/or chemical means to disrupt the BBB transiently for subsequent passage of therapeutics (especially small molecules) across the barrier [30]–[36]. These methods, however, have several limitations. For example, convection-enhanced delivery requires invasive procedures and can result in ineffective tissue distribution of the drugs injected. Intra-arterial injection of hyperosmolar agents such as mannitol causes reversible disruption of the BBB but the strategy is believed to cause lengthy disruption of the BBB and is also believed to cause significant expansion of the vascular volume. Drug delivery across the BBB by ultrasound generation of microbubbles is currently being investigated in several laboratories. Limitations of this method include controlling the size of the microbubbles, and preventing irreversible damage to blood vessels and endothelial cells [6]–[8]. Since lipid solubility enhances passive diffusion of a molecule across the BBB, several investigators have pursued such chemical modification (lipidization) to deliver drugs to the brain. However, lipidization is an expensive and time-consuming process, and the process itself may alter the pharmacokinetic properties of the drug [37], [38].

In this paper we demonstrate the ability of a synthetic peptide carrier, K16ApoE, to deliver eight different molecules (cisplatin, methotrexate, cetuximab, three different dyes, a synthetic peptide (Y8) and I-125) to the brain without requiring any chemical modification of the molecules. Brain delivery of the molecules is based on the premise that upon injection into the vasculature, K16ApoE binds to proteins in the blood creating apolipoprotein E (ApoE)-like entities. These entities are recognized by LDLR on the endothelial cell surface at the BBB as near-normal ligands and transcytosis is initiated. We further speculate that during ligand-receptor-mediated transcytosis transient pores are formed, which passively allow transport of other molecules to the brain. Since interaction of ApoE-like molecules with LDLR is an active process and since this interaction is speculated to create transient pores across the BBB that allow passive transport of non-ligand molecules, we use the term ‘actively-passive transport (APT)’ to describe this phenomenon. Conceptually and mechanistically, APT is likely an integral part of the BBB. Indeed, the brain-uptake of I-125 by insulin provides evidence of transient BBB permeability associated with ligand-receptor-based signaling intrinsic to the BBB. Similar data have been reported by Carman et al [39] that demonstrate BBB permeability as a consequence of AR signaling. Thus, APT is a two-step process: transcytosis of a ligand (or a ligand-like molecule such as K16ApoE-bound proteins) through interaction with its receptor at the BBB followed by transient permeabilization of the BBB as a result of transcytosis. We further speculate that most, if not all, ligand-receptor interactions that occur on the cell surface elicit APT probably even at non-BBB locations. At this time, we do not know if APT allows one-way (only to the brain) or two-way (to and from the brain) passage of molecules.

Before proceeding to explore delivery of cisplatin and methotrexate via K16ApoE, we tested K16ApoE-mediated brain-uptake with three dye molecules. No brain-uptake of the dyes was observed when the dyes were first mixed with K16ApoE and then injected. This result may be explained by the possibility that dye binding to K16ApoE blocked the ApoE moiety of the peptide. Thus the complex may have become inaccessible to the LDLR preventing transient opening of the BBB. Indeed, all the three dyes we have used are known to bind to proteins [40]–[42]. However, the fact that the dyes crossed the BBB when administered separately from the peptide illustrates a practical means to deliver such small molecules to the brain.

We have essentially developed three different APT approaches to delivering various potential drugs to the brain assisted by K16ApoE. In the first, agents that might bind to the transporter peptide and mask its ApoE moiety are delivered to the brain by separate injections of the drug and the peptide. In the second, agents that do not bind to the peptide can be delivered by mixing the two molecules and injecting only once. The third approach may be the most practical – this approach considers the likelihood that K16ApoE injected alone binds proteins (and other entities) in the blood, all of which could transcytose to the brain. This may be undesirable. To minimize such a possibility, K16ApoE can be pre-mixed with any desired protein (human serum albumin, for example) and used as the transporter. We mixed K16ApoE with cetuximab to illustrate that this approach can be adopted to deliver two anti-cancer drugs (such as cetuximab and cisplatin) simultaneously to the brain.

Direct intracranial delivery of a drug is routinely practiced in certain clinical situations. To be effective and acceptable as an alternative and relatively non-invasive means to deliver a drug to the brain, a method in question should allow comparable distribution of the drug in the brain to that obtained by intracranial injection. This premise was explored by delivering Evans Blue by both intracranial and K16ApoE-mediated methods. The results obtained provide a striking contrast in favor of the K16ApoE-mediated approach such that whereas EB was localized in a small area of the brain after intracranial delivery, the dye appeared to have a homogeneous distribution *throughout* the brain when delivered via K16ApoE, suggesting that the K16ApoE-based method is not only able to deliver a molecule to the brain, the method may be preferable over other options since it enables distribution of the molecule throughout the brain, which may be particularly desirable in the treatment of certain brain-associated disorders.

The BBB is practically a ‘closed door’ in the context of delivering therapeutics to the brain. It is known that receptors at the BBB provide a normal means for the transport of cognate ligands to the brain. Based on the results presented herein, coupled with the reports that the BBB can be transiently opened by activation of the adenosine receptor and endothelial cell B2 receptors by bradykinin [39], [43], [44], we propose that routine ligand/receptor binding also permits various other molecules to passively cross the barrier. Data presented in Table S1 suggest effective K16ApoE-mediated brain-delivery depends upon size. The effective brain-uptake by this method probably is also influenced by structure of a ‘cargo’ and other parameters implying that there could be certain molecules which will be resistant to brain-uptake by our method. Consequently, brain-uptake of a given molecule by our method must first be empirically determined before attempting to address its functional relevance to brain-associated parameter. In the future, molecules with defined structural variation without significantly changing molecular weight will need to be systematically created to adequately evaluate structure-activity relationship (SAR) relevant to the method to determine structural parameters of molecules that may have either positive or negative effect on the utility of the delivery method presented herein.

Our approach, in effect, mimics a normal physiological process, which might affect the BBB less than methods which utilize physical or chemical means to open the barrier. Since multiple ligand-receptor based systems are operational at the BBB, it is possible that many of them can be modeled to mimic our strategy. Thus, various combinations of such strategies may be employed to deliver a ‘larger’ amount of therapeutics, whenever necessary. It may also be possible to deliver a relatively greater amount of a drug to cross the BBB by employing a combination of our approach and inhibitor of efflux pumps [45], [46]. In addition, these methods may also be used to lower the conventional dose of BBB-penetrating therapeutics by enhancing their brain-uptake. Very recently, Siegal [47] has put forward five requirements that a chemotherapeutic or a brain-delivery strategy must fulfill to establish its potential to change clinical practice. As such, our method presented herein appears to fulfill three of the five requirements (feasibility of repeated or continuous administration, easy introduction into clinical practice and usefulness irrespective of size and location of a tumor in the brain). Whether our method fulfills the other two requirements (effectiveness and favorable adverse effects profile) will need to be investigated. Thus, future investigation will need to focus on evaluating clinical efficacy of the K16ApoE-mediated brain uptake of therapeutics in the management of patients with brain cancer and other brain-associated disorders. In this context, it is important to note that we have very recently demonstrated near-complete recovery of disease symptoms in a mouse model of Batten disease by K16ApoE-mediated delivery of recombinant tripeptidyl peptidase 1(TPP1) in TPP1 knockout mice [48], [49].

## Supporting Information

Figure S1 Brain uptake of cetuximab with and without K16ApoE. Different ratios of cetuximab and K16ApoE were used. One animal was evaluated for a given amount of cetuximab and K16ApoE.
(PPTX)

Table S1 Relationship between K16ApoE-mediated brain uptake of molecules and their size. Extent of brain uptake of four different molecules with molecular weights ranging from 125 to 1323 daltons are shown.
(DOCX)
