# Supplementary material for: Peptide Carrier-Mediated Non-Covalent Delivery of Unmodified Cisplatin, Methotrexate and Other Agents via Intravenous Route to the Brain
Source: PLoS One. 2014 May 21;9(5):e97655. doi: 10.1371/journal.pone.0097655 (PMC4029735; doi:10.1371/journal.pone.0097655)
Supplement: Figure S1 — Brain uptake of cetuximab with and without K16ApoE. Different ratios of cetuximab and K16ApoE were used. One animal was evaluated for a given amount of cetuximab and K16ApoE. (PPTX) [file pone.0097655.s001.pptx]

## Slide 1
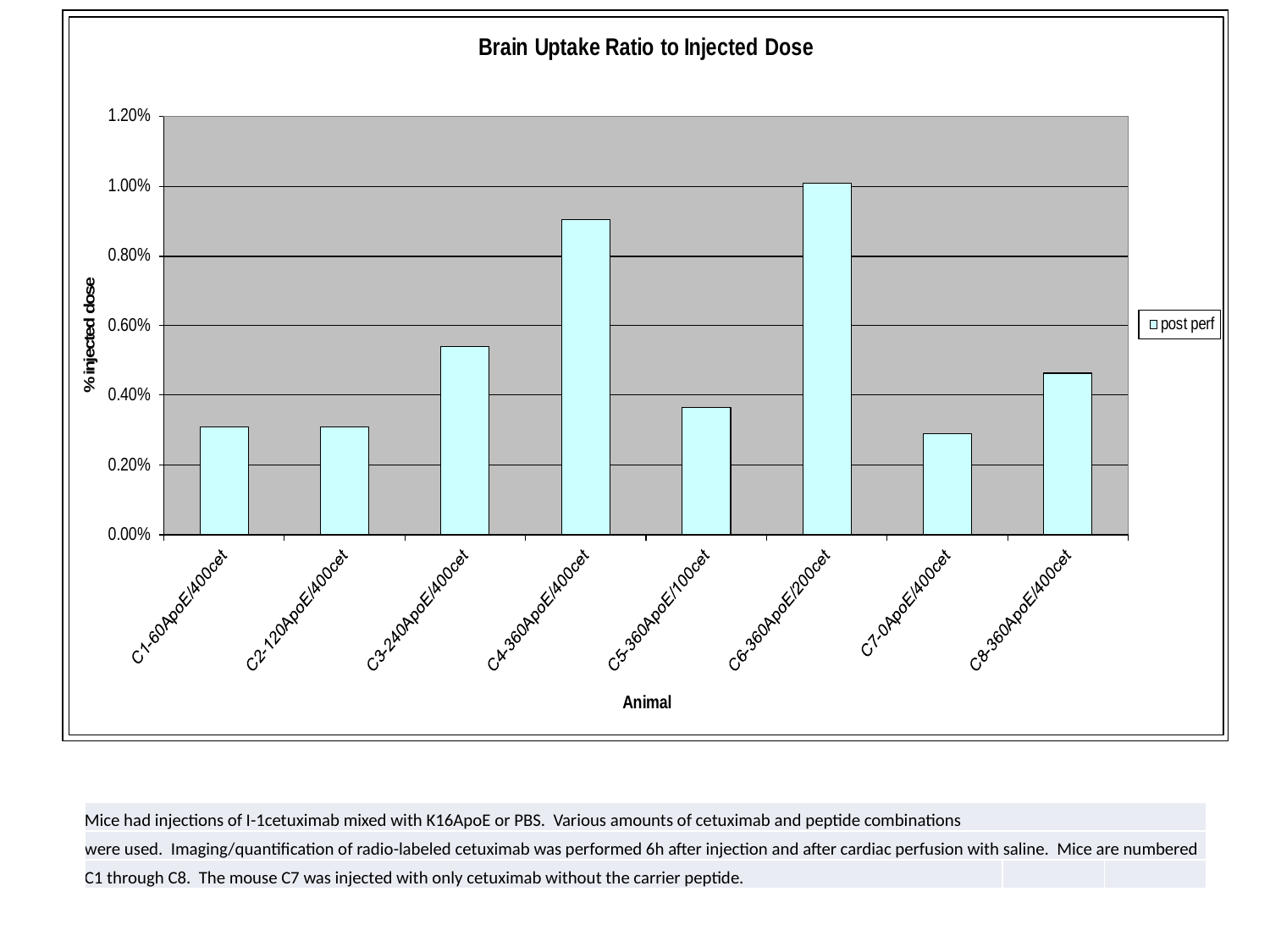

| Mice had injections of I-1cetuximab mixed with K16ApoE or PBS. Various amounts of cetuximab and peptide combinations | | |
| --- | --- | --- |
| were used. Imaging/quantification of radio-labeled cetuximab was performed 6h after injection and after cardiac perfusion with saline. Mice are numbered | | |
| C1 through C8. The mouse C7 was injected with only cetuximab without the carrier peptide. | | |
