# Supplementary material for: Peptide Carrier-Mediated Non-Covalent Delivery of Unmodified Cisplatin, Methotrexate and Other Agents via Intravenous Route to the Brain
Source: PLoS One. 2014 May 21;9(5):e97655. doi: 10.1371/journal.pone.0097655 (PMC4029735; doi:10.1371/journal.pone.0097655)
Supplement: Table S1 — Relationship between K16ApoE-mediated brain uptake of molecules and their size. Extent of brain uptake of four different molecules with molecular weights ranging from 125 to 1323 daltons are shown. (DOCX) [file pone.0097655.s002.docx]

Table S1: Relationship between brain-uptake of molecules and their size.

| Molecule | MW | % brain-uptake of injected dose |
| --- | --- | --- |
| Y8 | 1323 | 0.42 |
| Methotrexate | 454.45 | 0.54-0.92 |
| Cisplatin | 300.05 | 0.72-1.14 |
| I-125 | 125 | 1.52 |
